# Late-life learning and health: challenges, opportunities, and future directions

**DOI:** 10.1016/S2666-7568(21)00207-5

**Published:** 2021-10

**Authors:** Adina Zeki Al Hazzouri, Martine Elbejjani, Maya Abi Chahine, Ritu Sadana, Abla M Sibai

**Affiliations:** a Department of Epidemiology, Mailman School of Public Health, Columbia University, New York, NY, USA; b Department of Internal Medicine, Clinical Research Institute, American University of Beirut, Beirut 1107 2020, Lebanon; c University for Seniors Program, American University of Beirut, Beirut 1107 2020, Lebanon; d Department of Epidemiology, American University of Beirut, Beirut 1107 2020, Lebanon; e Ageing and Health, World Health Organization, Geneva, Switzerland

Population ageing is one of the most substantial demographic changes of the 21st century. At present, older generations are generally more educated, healthier, and are living longer than their parents, making them not only beneficiaries of but also active contributors to society. Opportunities that sustain older individuals' active participation and functional ability are gradually being incorporated into policies and programmes and are regarded as key elements in international charters such as the UN Madrid International Plan of Action on Ageing^1^ and the WHO Global Strategy and Ageing and Health,^2^ which have subsequently been expanded on in the WHO Decade of Healthy Ageing Baseline Report^3^ and UN Decade of Healthy Ageing 2021–2030.^4^ These frameworks and strategies universally recognise late-life learning opportunities as crucial for active and healthy ageing. Yet, late-life learning is seldom integrated into ageing-related policies, programmes, and research and is generally underfunded. In contrast to compelling evidence on the importance of formal education—typically completed by adulthood or early midlife—on almost every measure of health (eg, from subjective health to brain health and to life expectancy), little data exist on the benefits of late-life learning in the literature and within the current discourse of active and healthy ageing research.

Late-life learning is a necessary continuation of lifelong learning and non-formal late-life learning opportunities are one particularly promising option for learning at an older age. In contrast to formal learning that is directed towards including older individuals within a workforce, with the aim of credentialed professional advancement, non-formal educational opportunities are non-vocational and adopt a more holistic approach towards learning and growth by providing intellectually enriching experiences and social engagement opportunities, thereby enabling people to be and do what they value. These opportunities are aimed at enhancing the psychological wellbeing, social inclusion, and empowerment of older adult learners, thus having important links with frameworks of active and healthy ageing, particularly the ability to learn, grow, and make decisions.^3^ Several models of non-formal late-life learning exist around the world. Among the first to be established were the Institute for Retired Professionals programme in 1962, founded in association with the New School University (New York, NY, USA), and the University of the Third Age, which was established in 1972 at the University of Toulouse (Toulouse, France).^5^ Different models of the University of the Third Age were introduced in the UK in the early 1980s, which were less academically focused and emphasised the benefits of self-sufficiency, autonomy, and peer-to-peer learning. These early interventions provided a starting point for the replication of similar initiatives elsewhere in Europe and around the world. Late-life learning efforts have progressed substantially since their inception in the 1960s with programmes and offerings that operate under various hybrid models and titles (eg, University for Seniors, Learning in Retirement, Senior Club, Senior Center, Study Circle).

Despite the growing interest in late-life learning, formal assessments of late-life learning programmes and empirical data on their benefits and underlying causal mechanisms remain scarce. Furthermore, the little research available is mostly from high-income countries.^3^ Generally, appraisals of late-life learning programmes and their implications are limited by the wide variations in study contexts and research landscape, including type and organisational structures of the late-life learning programme (eg, university or community based; professional-led or peer-to-peer learning), programme framework, aims and goals, focus and content (eg, intellectual, social, physical, other, or a combination), and differences in study population and sample selection. Despite these limitations, gerontological theories and available data have highlighted two principal themes underlying the benefits of late-life learning: cognitive stimulation and social engagement and inclusion. Data from a small number of studies on the topic suggest that continued learning in older age can have a linked effect on public health^6^, ^7^, ^8^, ^9^ by raising health awareness and promoting better health practices and outcomes; providing multidimensional health benefits, including but not limited to, maintaining cognitive, mental, and overall wellbeing; building social capital and promoting motivation, social cohesion, and inclusiveness; and re-integrating and empowering older adults to withstand later-life challenges in increasingly complex realities of social isolation and loneliness, the importance of which has been highlighted during the COVID-19 pandemic.

Late-life learning programmes offer a unique platform to address exciting innovative research questions, and improve our understanding of the determinants of active and healthy ageing and inform future investments and the design of late-life learning programmes. Such programmes could serve as starting point for assessment of whether late-life learning can be built around targeted interventions for specific ageing-related disorders, such as dementia and declines in cognitive function, and hence provide a new and rare opportunity to modify the course of ageing-related pathologies. Research on the potential multidimensional health impact of late-life learning (ie, mental, emotional, and physical health, and overall wellbeing benefits) would contribute to strengthening the rationale, advocacy, and resources for the mainstreaming of late-life learning in national strategies and inform policies and intervention programmes. Notably, late-life learning programmes are inexpensive and rely on voluntary contributions from lecturers and tutors and thus are easily scalable; this makes such programmes particularly feasible in low-resource settings. Implementation research is needed to identify factors that would increase the reach of late-life learning to the disadvantaged and vulnerable groups and so-called fourth agers who are often challenged with declining health, frailty, and physical limitations. Due to the COVID-19 pandemic, a global shift to online learning has been observed, which could present a unique opportunity to promote the digital inclusion of older adults and attract a more equitable constituency of participants within a wider research and policy landscape.

We declare no competing interests. AZ and ME are joint first authors.
